# Obstructive Jaundice due to Pancreatic Involvement as an Initial Presentation of Adult Acute Lymphoblastic Leukemia

**DOI:** 10.1155/2018/9175360

**Published:** 2018-12-04

**Authors:** Oreoluwa Oladiran, Ifeanyi Nwosu

**Affiliations:** ^1^Reading Hospital, Tower Health System, West Reading, PA, USA; ^2^Leighton Hospital NHS Trust, Crewe, Cheshire, UK

## Abstract

Acute lymphoblastic leukemia (ALL) is a heterogeneous group of lymphoid disorders characterized by monoclonal proliferation and expansion of immature lymphoid cells in the bone marrow, blood, and other organs. It commonly presents with nonspecific symptoms such as lethargy, easy bruising, and weight loss. In this article, we present the case of a 48-year-old male who presented to the hospital with painless jaundice resulting from pancreatic infiltration, initially thought to be due to pancreatic or hepatobiliary malignancy. He was later diagnosed with ALL by lymph node biopsy and peripheral blood flow cytometry immunophenotyping and was transferred to a cancer treatment centre for unilateral bone marrow biopsy and further management. Our case highlights the rare occurrence of pancreatic infiltration in ALL.

## 1. Introduction

Acute lymphoblastic leukemia is a malignant neoplasm characterized by the proliferation of immature lymphoid cells in the bone marrow, peripheral blood, or other organs including the liver, pancreas, and kidneys. It is the commonest cancer among children and most frequent cause of cancer mortality in children younger than 20 years [1, 2]. It has an overall incidence of about 1.7 per 100,000 persons with a bimodal distribution [3]. An early peak occurs at age 4–5 years with an incidence of 4–5/100,000 persons, followed by a second gradual increase at about age 50 years with incidence up to 2/100,000 persons [4]. The 5-year overall survival rate is about 90% in children compared to 30% survival rate in older patients [4, 5]. Clinical features are protean and include fatigue, dyspnea, bone pain, easy bruising, and dizziness including B symptoms such as fever, weight loss, and night sweats. Physical findings commonly include pallor, ecchymosis, petechiae, lymphadenopathy, and splenomegaly. So far, only few cases of obstructive jaundice resulting from leukemic infiltration of the pancreas in acute lymphoblastic leukemia have been reported in literature [6, 7].

## 2. Case History

**Figure 1:**
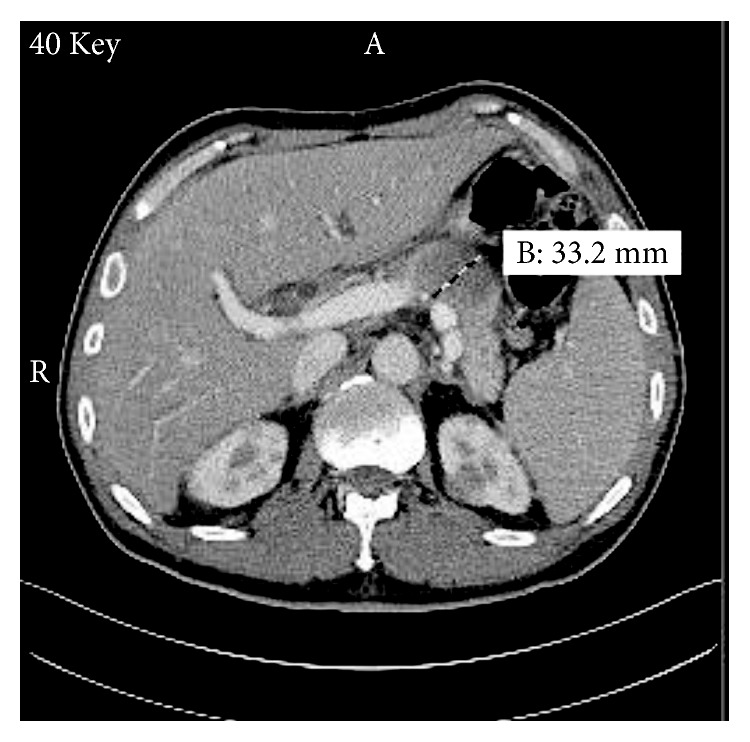
Computed tomography scan of the abdomen showing 33.2 mm mass in the pancreatic body. Multiple bilateral renal masses can also be appreciated.

A 48-year-old man with no significant past medical history presents to the emergency department with 1 week history of worsening jaundice associated with passage of dark coloured urine and pale stools. He denied abdominal pain, fever, chills, rigors, or night sweats. He had no skin rashes, pruritus, or easy bruising. He also reported no anorexia or unintentional weight loss. He was a nonsmoker and a nonalcoholic with no family history of malignancy. Vital signs were normal and physical examination was positive only for generalized icterus. He had no peripheral lymphadenopathy. Complete blood count revealed pancytopenia with white blood cell count of 3200/ml (normal 4800–10800/ml), hemoglobin of 13.8 g/dL (normal 14.0–17.5 g/dL), and platelet count of 71000/ml (normal 130000–400000/ml). Basic metabolic panel, including renal function test, was normal. Liver function test revealed alkaline phosphatase of 147 U/L (normal 34–104 U/L), aspartate transaminase of 65 U/L (normal 13–39 U/L), alanine transaminase of 190 U/L (normal 7–52 U/L), total bilirubin of 7.2 mg/dL (normal 0.3–1.0 mg/dL), and direct bilirubin of 4.3 mg/dL (normal <0.2 mg/dL). Abdominal ultrasound scan revealed 4.5 cm hypoechoic mass adjacent to the pancreatic tail with mild splenomegaly. It also reported mild dilatation of the common bile duct without sonographic evidence of acute cholecystitis. Computed tomography (CT) scan of the abdomen and pelvis with contrast (Figure 1) revealed 36 × 33 mm mass-like thickening of the pancreatic body with a nodular mass contiguous with the pancreatic head and multiple renal masses. CT scans of the head and chest, done to exclude presence of other primary source or metastatic disease, were normal.

Based on the history of painless obstructive jaundice and the imaging findings above, our initial suspicion was pancreatic or bile duct malignancy. IgG4-related disease was also considered in the differential diagnosis. Autoimmune diseases may also present as painless jaundice but less common in males with no family history of autoimmune disease. Interestingly, carcinoembryonic antigen (CEA) was 1.4 ng/mL (normal <3 ng/mL) and CA 19–9 was 28 U/mL (normal <37 U/mL). IgG4 level was 38 mg/dL (normal 1–123 mg/dL).

He was planned for diagnostic esophagogastroduodenoscopy, endoscopic ultrasound scan, and fine needle aspiration which revealed multiple enlarged hypoechoic homogenous malignant appearing lymph nodes within the peripancreatic, perihepatic, and periportal regions. Biopsy confirmed precursor B acute lymphoblastic lymphoma. Peripheral blood flow cytometry also revealed mixture of cells with lymphoid predominance. A monoclonal population of blasts coexpressing CD10/CD19/CD20/CD22/CD38/HLA-DR/CD79a and TdT was noted. CD34 expression was not seen. Cytogenetic studies were negative for deletion of CDKN2A (p16), rearrangement of KMT2A (MLL) and TCRA/D, and translocation of ETV6/RUNX1 and BCR/ABL1. This phenotypic expression was consistent with B-cell lymphoblastic lymphoma, and a diagnosis of acute lymphoblastic lymphoma (ALL) was made. He was promptly transferred to the regional cancer centre for further evaluation and treatment. At the cancer centre, his liver enzymes worsened and he underwent endoscopic retrograde cholangiopancreatography (ERCP) which revealed a stricture at the common bile duct (CBD) and underwent sphincterotomy and stent deployment and gradual resolution of deranged liver enzymes. He consented to enrolment in a clinical trial and was commenced on daunorubicin, vincristine, methotrexate, cyclophosphamide, rituximab, pegaspargase, and intrathecal cytarabine. He was discharged home in the stable condition after 2 weeks and planned for weekly intrathecal chemotherapy and follow-up with the oncologist.

## 3. Discussion

Our patient was diagnosed with acute lymphoblastic leukemia (ALL). Typical symptoms include the B symptoms such as fever, unintentional weight loss, and night sweat. Recurrent infections, easy bruisability, bleeding, fatigue, and bone pain reflect the progressive bone marrow failure and expansion of the marrow by immature blast cells [9, 13]. Extramedullary involvement in ALL is not unusual with the leukemic cells commonly infiltrating the central nervous system, testes, lymph nodes, and spleen [8]. It is believed that the overexpression of CXCR4, a chemokine receptor, on abnormal leukemic lymphoblasts is associated with extramedullary infiltration. These receptors bind to the chemokine stromal-cell-derived factor-1 (SDF-1), a potent molecule for chemotaxis of lymphocytes found in the bone marrow and other tissues [14]. Rare cases of pancreatic and hepatic involvement with extrahepatic and intrahepatic bile duct obstruction causing cholestatic jaundice as an initial presentation have also been reported in adult ALL [6, 7].

Adult ALL is a less common haematological malignancy making up about 20% of acute leukemias in adult, with a poor survival rate of 30–40% compared to the higher survival rate of about 90% in paediatric ALL [8]. Three subtypes exist based on the morphology and cytogenetic profile as described by the World Health Organisation (WHO)-B lymphoblastic, T lymphoblastic, and the Burkitt leukemia. The B-cell lymphoblastic leukemia accounts for about 75% of adult ALL [9]. Earlier classification system by French American British (FAB) based purely on morphology of the leukemic cells is now considered obsolete. The exact cause is unknown, but multiple genetic conditions and environmental exposures are known to increase the risk of developing ALL. Incidence is higher in patients with Down syndrome with a 20-fold increase in risk, Fanconi anemia, Bloom syndrome, and ataxia telangiectasia as well as environmental exposure to benzene products, ionizing radiations, and viruses including Epstein–Barr virus and human immunodeficiency virus [9, 10]. Common cytogenetic abnormalities in the form of chromosomal translocation seen in ALL include *t*(9; 22), *t*(4; 11), and *t*(12; 21). Adult ALL is associated with *t*(9; 22) (q34; q11) (Philadelphia chromosome) with the BCR-ABL1 fusion in 15–30% representing the most frequent molecular abnormality [11]. This is in contrast to paediatric ALL where *t* (12; 21) (p13, q22) is the most common chromosomal translocation and carries a favourable prognosis [12]. Presence of Philadelphia chromosome is believed to be a poor prognostic feature and is seen more in adult ALL [11].

Our patient had both pancreatic and bilateral renal involvements, and his obstructive jaundice was believed to be secondary to the pancreatic masses as evidenced by the mildly dilated CBD without any obvious hepatic or gall bladder pathologies. Involvement of the pancreas in ALL is very rare and even more uncommon is the concurrent renal involvement [15]. Only few publications exist for adult ALL presenting primarily with obstructive jaundice secondary to pancreatic infiltration by leukemic cells. Daniel et al. reported the first case of obstructive jaundice from pancreatic masses compressing the common bile duct (CBD) in adult ALL [7]. Another rare case of leukemic infiltration of hepatic sinusoids resulting in cholestasis without pancreatic involvement was described by Siddique et al. [6].

The differential diagnoses of jaundice are broad, sometimes presenting a diagnostic dilemma as seen in our case where the initial suspicion was hepatobiliary or pancreatic malignancy. It is therefore prudent for clinicians to consider this possibility during initial diagnostic workup of patients presenting with obstructive jaundice to forestall unnecessary invasive investigations. Obstructive jaundice in ALL may also pose a challenge during initiation of treatment because most chemotherapeutic agents are metabolized by the liver; however, if liver dysfunction is directly attributable to ALL, judicious use of cytoreductive treatment with selected agents may lead to improved liver function and thus facilitate safer administration of definitive induction therapy [16].

## References

[1] (July 2018). Cancer statistics review, 1975-2013-previous version-SEER cancer statistics review. https://seer.cancer.gov/archive/csr/1975_2013/.

[2] Jemal A., Tiwari R. C., Murray T. (2004). Cancer statistics, 2004. *CA: A Cancer Journal for Clinicians*.

[3] Paul S., Kantarjian H., Jabbour E. J. (2016). Adult acute lymphoblastic leukemia. *Mayo Clinic Proceedings*.

[4] Jabbour E., O’Brien S., Konopleva M., Kantarjian H. (2015). New insights into the pathophysiology and therapy of adult acute lymphoblastic leukemia. *Cancer*.

[5] Alvarnas J. C., Brown P. A., Aoun P. (2015). Acute lymphoblastic leukemia, version 2.2015. *Journal of the National Comprehensive Cancer Network*.

[6] Siddique M. N., Popalzai M., Aoun N., Maroun R., Awasum M., Dai Q. (2011). Precursor B-cell acute lymphoblastic leukemia presenting as obstructive jaundice: a case report. *Journal of Medical Case Reports*.

[7] Daniel S. V., Vani D. H., Smith A. M., Hill Q. A., Menon K. V. (2010). Obstructive jaundice due to a pancreatic mass: a rare presentation of acute lymphoblastic leukaemia in an adult. *Journal of Oncology Practice*.

[8] Heincelman M., Karakala N., Rockey D. C. (2016). Acute lymphoblastic leukemia in a young adult presenting as hepatitis and acute kidney injury. *Journal of Investigative Medicine High Impact Case Reports*.

[9] Terwilliger T., Abdul-Hay M. (2017). Acute lymphoblastic leukemia: a comprehensive review and 2017 update. *Blood Cancer Journal*.

[10] Hasle H., Clemmensen I. H., Mikkelsen M. (2000). Risks of leukaemia and solid tumours in individuals with Down’s syndrome. *The Lancet*.

[11] Faderl S., O’Brien S., Pui C.-H. (2010). Adult acute lymphoblastic leukemia. *Cancer*.

[12] Somanath P., RajLaxmi S., Pranati M., Das R., Sukumar C., Raghumani M. (2011). Pattern of chromosomal abnormalities in pediatric acute lymphoblastic leukemia (ALL). *Kuwait Medical Journal*.

[13] PDQ Adult Treatment Editorial Board PATE (2002). *Adult Acute Lymphoblastic Leukemia Treatment (PDQ®), Health Professional Version*.

[14] Skeith L., Lazo-Langner A., Mangel J. (2013). Kidney and pancreatic extramedullary relapse in adult acute lymphoblastic leukemia: a case report and review of the literature. *Case reports in Hematology*.

[15] Ramanathan S., Prakash M., Khandelwal N. (2014). Concurrent pancreatic and renal leukemic cell infiltration. *Indian Journal of Hematology and Blood Transfusion*.

[16] Potosky D., Twaddell W., Khurana S. (2010). Image of the month. Acute lymphoblastic leukemia presenting as painless jaundice. *Clinical Gastroenterology and Hepatology*.

